# Perspectives in immunotherapy: meeting report from the Immunotherapy Bridge (29-30 November, 2017, Naples, Italy)

**DOI:** 10.1186/s40425-018-0377-z

**Published:** 2018-07-11

**Authors:** Paolo A. Ascierto, James Brugarolas, Luigi Buonaguro, Lisa H. Butterfield, David Carbone, Bruno Daniele, Robert Ferris, Bernard A. Fox, Jérôme Galon, Cesare Gridelli, Howard L. Kaufman, Christopher A. Klebanoff, Ignacio Melero, Paul Nathan, Chrystal M. Paulos, Marco Ruella, Ryan Sullivan, Hassane Zarour, Igor Puzanov

**Affiliations:** 1 Melanoma, Cancer Immunotherapy and Development Therapeutics Oncology Unit, Istituto Nazionale Tumori IRCCS Fondazione “G. Pascale, Napoli, Italy; 2 Kidney Cancer Program, Department of Internal Medicine, Harold C. Simmons Comprehensive Cancer Center, University of Texas Southwestern Medical Center, Dallas, Texas USA; 3 Molecular Biology and Viral Oncology Unit, Istituto Nazionale Tumori IRCCS Fondazione “G. Pascale, Napoli, Italy; 4 UPCI Immunologic Monitoring and Cellular Products Laboratory, University of Pittsburgh, Pittsburgh, Pennsylvania USA; 5 College of Medicine, James Thoracic Center, James Cancer Hospital and Solove Research Institute, The Ohio State University, Columbus, Ohio USA; 6 Department of Oncology, “G. Rummo” Hospital, Benevento, Italy; 7 Division of Head and Neck Surgery, Department of Otolaryngology, University of Pittsburgh, Pittsburgh, Pennsylvania USA; 8 Laboratory of Molecular and Tumor Immunology, Robert W. Franz Cancer Research Center in the Earle A. Chiles Research Institute at Providence Cancer Center, Portland, Oregon USA; 9 National Institute of Health and Medical Research (INSERM), Paris, France; 10 Unit of Medical Oncology, Hospital “San Giuseppe Moscati”, Avellino, Italy; 11 Robert Wood Johnson Medical School Rutgers, The State University of New Jersey, New Brunswick, New Jersey USA; 12 Center for Cell Engineering and Department of Medicine, Memorial Sloan Kettering Cancer Center, New York, New York USA; 13 Immunology and Immunotherapy Service, Clinica Universidad de Navarra, Pamplona, Navarra Spain; 14 Mount Vernon Cancer Centre, Northwood, Middlesex UK; 15 Department of Microbiology and Immunology Hollings Cancer Center, Medical University of South Carolina (MUSC), Charleston, South Carolina USA; 16 Center for Cellular Immunotherapies, University of Pennsylvania, Philadelphia, Pennsylvania USA; 17 Medicine Harvard Medical School and Haematology/Oncology Department, Massachusetts General Hospital, Boston, Massachusetts USA; 18 Melanoma Program, University of Pittsburgh Cancer Institute, Pittsburgh, Pennsylvania USA; 19 Early Phase Clinical Trials Program, Experimental Therapeutics Program, Melanoma Section, Department of Medicine, Roswell Park Cancer Institute, Buffalo, New York USA

**Keywords:** Immunotherapy, Checkpoint inhibitors, Cancer vaccines, Adoptive cell transfer combination therapy, Biomarkers

## Abstract

Immunotherapy represents the third important wave in the history of the systemic treatment of cancer after chemotherapy and targeted therapy and is now established as a potent and effective treatment option across several cancer types. The clinical success of anti-cytotoxic T-lymphocyte-associated antigen (CTLA)-4, first, and anti-programmed death (PD)-1/PD-ligand (L)1 agents in melanoma and other cancers a few years later, has encouraged increasing focus on the development of other immunotherapies (e.g. monoclonal antibodies with other immune targets, adoptive cell transfer, and vaccines), with over 3000 immuno-oncology trials ongoing, involving hundreds of research institutes across the globe. The potential use of these different immunotherapeutic options in various combinations with one another and with other treatment modalities is an area of particular promise. The third Immunotherapy Bridge meeting (29-30 November, 2017, Naples, Italy) focused on recent advances in immunotherapy across various cancer types and is summarised in this report.

## Background

Immunotherapy represents the third important wave in the history of the systemic treatment of cancer, following on from the advent of chemotherapy in the 1940’s and targeted therapy in the late 1990’s. Since its first clinical application as Coley's toxins towards the end of the 19^th^ century, after a postsurgical infection was observed to result in spontaneous tumour regression, the field of immunotherapy has finally come of age and is now established as a potent and effective treatment option across several cancer types. The clinical success of immune checkpoint blockade with anti-cytotoxic T-lymphocyte-associated antigen (CTLA)-4 and anti-programmed death (PD)-1/PD-ligand (L)1 agents in melanoma and other cancers has encouraged increasing focus on the development of other immunotherapies, particularly monoclonal antibodies with other immune targets, adoptive cell transfer and vaccines. Indeed, it has been estimated that there are over 3000 immuno-oncology trials ongoing, targeting hundreds of disease and immune pathways and involving hundreds of research institutes across the globe. The potential use of these different immunotherapeutic options in various combinations with one another and with other treatment modalities is an area of particular promise. This report summarizes the recent advances in immunotherapy across various cancer types as discussed during the third Immunotherapy Bridge meeting (29-30 November, 2017, Naples, Italy).

## Cumulative suppression index, cancer vaccines and a strategy to develop combination immunotherapy with T cell agonists

Evaluation of T lymphocyte frequency provides prognostic information for patients with cancer. Moreover, the location and relative positions between immune populations (i.e. distance of T regulatory cells [Tregs] and PD-L1 to CD8 T cells) are important factors in understanding their function in a complex environment and this information can enhance the prognostic power of CD8^+^ cells. Integrating this information into a cumulative suppression index (CSI) can increase correlation with overall survival (OS) and incorporating tumour expression levels of antigen-processing machinery components can further improve prognostic power [1]. If validated, CSI may be useful in stratifying patients for clinical trials as well as directing therapy choices.

Autophagy is a cellular process in which portions of the cytoplasm are sequestered by double membrane vesicles termed autophagosomes and is essential for efficient cross-presentation and subsequent induction of tumour immunity. Cross-presentation is significantly inhibited when autophagy is blocked and increased when autophagy is promoted. Isolated autophagosome-containing vesicles, known as Dribbles can serve as a potent antigen source and have shown cross-protection against related tumours and efficacy against established tumours in preclinical studies [2]. Efficacy may be via presentation of short-lived proteins (SLiPs) and defective ribosomal products (DRiPs) normally not cross-presented by antigen-presenting cells. DPV-001 is a DC-targeted (CLEC9A) microvesicle vaccine derived from an adenocarcinoma and a mixed histology cell line that contains multiple toll-like receptor (TLR) agonists and >130 potential non-small cell lung cancer (NSCLC) antigens, many as prospective altered-peptide ligands. In a phase II trial, DPV-001 alone or with granulocyte-macrophage colony-stimulating factor (GM-CSF) or imiquimod for adjuvant treatment of stage III NSCLC was tolerable and induced or boosted IgG antibodies to TAAs (tumour-associated antigens) [3]. DPV-001 also expanded populations of T cells with increases in CD4 T cells similar to those observed in patients receiving anti-CTLA-4 ipilimumab [4].

DPV-001 is also being evaluated in combination with anti-OX40 agonists. OX-40 increases T cell expansion and cytokine production and OX-40 signalling also controls regulatory T cell differentiation and suppressive function [5]. Although OX-40 agonists enhance anti-tumour immunity in immunogenic tumours, poorly immunogenic tumours are less responsive. Combining vaccine strategies that prime tumour-specific T cells with OX-40 agonists could sustain anti-tumour responses.

## Development of cancer vaccines for hepatocellular carcinoma

Hepatocellular carcinoma (HCC) accounts for about 6% of all new cancers worldwide and represents the third most common cause of cancer-related death. The overall prognosis for patients with HCC is poor, especially in patients with more advanced disease stage in which available treatments (*e.g.* sorafenib, an anti-vascular endothelial growth factor inhibitor) have limited efficacy. Immunotherapy-based strategies may represent a novel and effective tool for patients with HCC, although previous efforts have had only mixed success.

One potential immunotherapeutic approach in HCC is the development of peptide vaccines. Tumour-associated antigens (TAAs) are self-derived proteins rendered immunogenic in tumours by aberrant expression. In HCC patients, several TAAs can spontaneously induce CD8^+^ T cell responses including alpha fetoprotein (AFP), glypican-3 (GPC-3), and melanoma-associated gene-A1 (MAGE-A1). The first HCC vaccine clinical trial was based on CD8^+^ T cell epitopes specific for AFP and showed T cell responses in vaccinated subjects [6]. The same group performed a subsequent phase I/II trial administering AFP epitopes presented by autologous dendritic cells (DCs) loaded *ex vivo*. This, however, only produced transient CD8^+^ T cell responses, possibly due to the lack of CD4^+^ help [7, 8]. To increase the number of TAAs targeted by the immune response, vaccines based on autologous DCs pulsed *ex vivo* with a lysate of the autologous tumour [9] or hepatoblastoma cell line HepG2 [10, 11] were evaluated, but achieved only limited improvements in clinical outcomes. Other trials, including low-dose cyclophosphamide treatment followed by a telomerase peptide (GV1001) vaccination [12], MRP3-derived peptide (MRP3765) [13] and adjuvant GPC-3 peptide [14] vaccine have also had mixed results.

The main limiting factors in HCC vaccine development is that the TAAs used in clinical trials are limited in number and not HCC-specific, together with the inherent intra-hepatic immunosuppressive environment. The current ongoing EU-funded HepaVAC project is developing a new concept of therapeutic cancer vaccines for HCC, aimed at overcoming the limitations of previous efforts (www.hepavac.eu). The main goal of HepaVAC is to develop a novel therapeutic cancer vaccine to improve clinical outcome after standard therapy. The HepaVac vaccine consists of an ‘off-the-shelf’ vaccine comprising 18 newly identified MHC-I and II tumour-associated peptides (TUMAPs) naturally processed and presented on primary tumour tissues from HCC patients (HLA peptidome), for the induction of tumour-specific CD4^+^ T helper cell and cytotoxic CD8^+^ lymphocyte effector and memory immune responses. In a subgroup of enrolled patients, an actively personalised vaccine (APVAC) will be administered during the treatment as boosting antigen, based on patient-specific mutated and naturally processed and presented peptides. Both vaccines will be combined with a novel and potent RNA-based immunomodulator [15]. As part of this initiative, a first-in-man, open-label, multicentre European phase I/II clinical trial (HepaVac-101; NCT03203005) will assess the safety, tolerability and immunogenicity of the vaccine. To date, five of six study sites have initiated the trial and started screening patients.

A related EU-supported project is HEPAMUT, the primary aim of which is the identification and immunological validation of mutated neoantigens specific to HCC (www.hepamut.eu). This project will involve evaluating the HCC mutanome and predicting the presentation of neoepitopes by HLA-A2*01 allele, assessing the frequency of specific T cells to such mutant epitopes in HCC patients, and validating the immunogenicity of neoepitopes in HLA-transgenic mice and their therapeutic effect in a humanised patient-derived xenograft mouse model.

One important consideration in the identification of neoantigens is the distinction between true and false neo-antigens. Mutated peptides may represent non-self neoantigens that are exclusively presented on tumour cells and are not affected by central T cell tolerance. In an analysis of tumour tissue from patients with melanoma treated with anti-CTLA-4 ipilimumab or tremelimumab, whole-exome sequencing revealed a neoantigen landscape specifically present in tumours with a strong response to CTLA-4 blockade, with the presence of specific tumour neoantigens shared by patients with long-term clinical benefit but absent in patients with minimal or no benefit [16]. Data suggest that the neoepitopes in patients with strong clinical benefit from CTLA-4 blockade may resemble epitopes from pathogens that T cells are likely to recognise. Thus, patients with neoantigens similar to pathogen antigens are more likely to respond to treatment. False predictive neoantigens have similar predicted antigenicity to the corresponding wild-type epitope and may be less likely to confer benefit.

## Novel combinatorial immunotherapies with PD-1 blockade from the bench into the clinic

**Fig. 1:**
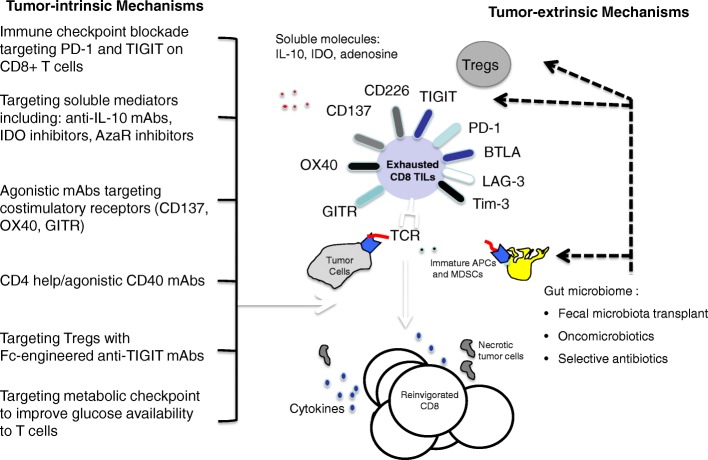
Therapeutic strategies to target tumour-intrinsic and tumour-extrinsic mechanisms driving anti-tumour T cell dysfunction

Anti-PD-1 antibodies represent a potent therapy of melanoma and other solid tumours. However, resistance to PD-1 blockade is an ongoing problem and various other strategies to target tumour-intrinsic and tumour-extrinsic mechanisms driving anti-tumour T cell dysfunction are being assessed (Fig. 1). Two targets for immune checkpoint blockade are T cell immunoglobulin domain and mucin domain-3 (Tim-3) and T cell Immunoglobulin and ITIM domain (TIGIT). Dual Tim-3 and PD-1 expression is associated with enhanced tumour antigen-specific CD8^+^ T cell dysfunction in melanoma patients [17]. TIGIT is also upregulated on tumour antigen-specific CD8^+^ T cells and CD8^+^ tumour-infiltrating lymphocytes (TILs) from patients with melanoma. These TIGIT-expressing CD8^+^ T cells often co-express PD-1 [18]. TIGIT ligands are highly expressed in metastatic melanoma and TIGIT and PD-1 blockade increases the proliferation, cytokine production, and degranulation of both tumour antigen-specific CD8^+^ T cells and CD8^+^ TILs in the presence of TIGIT ligand-expressing cells. CD8^+^ TILs exhibited downregulation of the costimulatory molecule CD226, which competes with TIGIT for the same ligand, supporting a TIGIT/CD226 imbalance in metastatic melanoma [18].

Dual PD-1/TIGIT blockade and dual PD-1/Tim-3 blockade are both potential strategies that are being assessed in metastatic melanoma. TSR-022 (Tesaro), an anti-TIM-3 monoclonal antibody, is being assessed alone and in combination with an anti-PD-1 antibody in a first-in-man dose escalation and cohort expansion phase I study of patients with advanced solid tumours (NCT02817633). Similarly, the anti-TIGIT antibody BMS 9862017 is being investigated in a phase I/IIa first-in-human study alone and in combination with anti-PD-1 nivolumab in advanced solid tumours (NCT02913313).

Therapeutic strategies that target tumour-extrinsic mechanisms driving anti-tumour T cell dysfunction are also being explored. One such example is faecal microbiota transplant (FMT). Gut microbiota from melanoma patients who respond to PD-1 inhibition have higher alpha-diversity and increased number of certain bacterial commensals as compared to PD-1 non-responders. In addition, FMT obtained from PD-1 responder melanoma patients appeared to convert PD-1 refractory mice with melanoma into PD-1 responders. A phase II feasibility study of FMT in PD-1 resistant melanoma is planned at the University of Pittsburgh to test the capability of the gut microbiome to modulate clinical responses to anti-PD-1 pembrolizumab in PD-1 refractory melanoma patients (NCT03341143).

## The search for blood-based biomarkers to predict immunotherapy outcomes

**Fig. 2:**
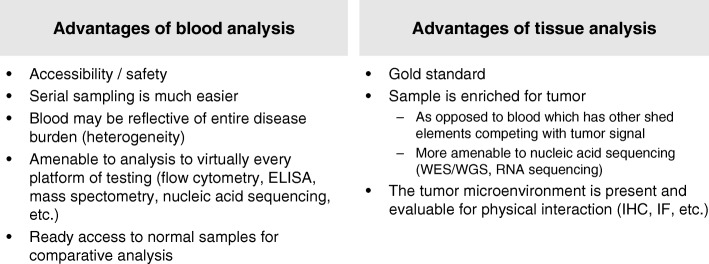
Blood-based biomarker development: blood versus tissue samples

PD-1/PD-L1 expression, cytolytic activity, and mutational load are positive and interdependent prognostic features in melanoma and other tumours [19]. Biomarkers for anti-PD-1/PD-L1 are required and blood-based markers offer several advantages over tissue-based markers, in that analysis may be easier and safer to perform, and blood may be indicative of the entire disease burden (Fig. 2). Blood samples are also amenable to virtually every analysis platform and allow ready access to normal samples for comparative analysis.

Circulating factors are likely to represent what is happening in the tumour. Circulating tumour DNA (ctDNA) isolated from plasma has been shown to reflect the mutational status of glioblastoma, and extracellular vesicles containing ctDNA, microRNA and proteins act as reservoirs for biomarkers such as typical DNA mutations, regulatory microRNAs and oncoproteins [20]. At Massachusetts General Hospital, serial ctDNA BRAF mutation has been shown to correlate with response/progression in patients treated with anti-BRAF vemurafenib and high-dose interleukin (IL)-2. Also, serial ctDNA mutation correlates with response/progression in patients treated with immune checkpoint inhibitors. Longitudinal assessment of ctDNA predicts response to anti-PD-1 antibodies in metastatic melanoma [21]. Non-detection or loss of detection of ctDNA was associated with excellent outcomes. Mutational load and possibly copy number alterations also predict response to immunotherapy; analysis of mutational load and copy number gains/losses is feasible from ctDNA. Genetic alterations associated with anti-PD-1 inhibitor resistance (*e.g.* B2M) are detectable in ctDNA using ultra-low pass whole genome sequencing and droplet digital polymerase chain reaction (ddPCR) [22].

Exosomes represent another potential biomarker source. Exosomes are extracellular vesicles that express a sub-proteome of the cell and encapsulate mRNA that can be transferred to other cells to modulate the recipients’ transcriptome [23]. Concordance in patient tumours has shown enrichment for immune pathways in patient plasma exosomes.

Circulating tumour cells (CTCs), exosomal RNA and serum protein profile also represent potential blood-based biomarkers for patients treated with anti-PD-1 therapy. New technologies and novel platforms are available to perform these broad and potentially high-impact analyses and the next steps are individual and cross-validation of these approaches.

## Genomics and immunotherapy in lung cancer: tumour mutation burden, mutations affecting antigen presentation, immune recognition, and genome integrity

**Fig. 3:**
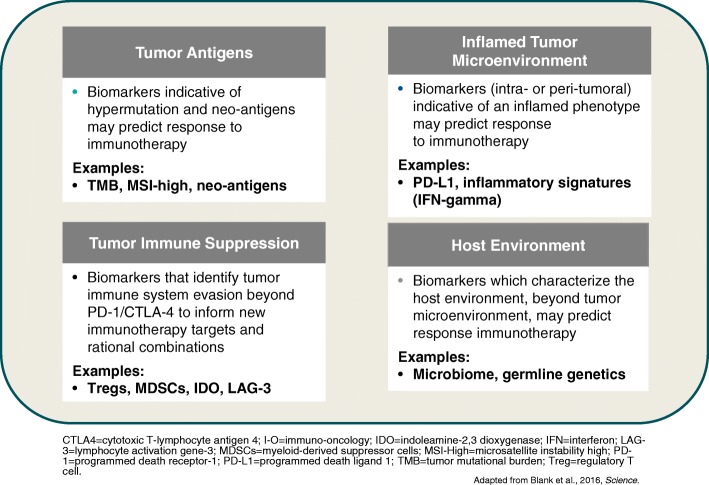
Tumour and Immune biomarkers being evaluated to predict better outcomes to immunotherapy

A wide range of tumour and immune biomarkers are being evaluated to predict better outcomes to immunotherapy (Fig. 3). High tumour mutation burden (TMB) may influence the immune-mediated anti-tumour response, meaning tumours with high TMB such as lung cancer are a rational target for treatment with immunotherapy. Studies have suggested that TMB may be a predictive biomarker for immunotherapy agents [16, 24]. In the CheckMate-026 trial, first-line nivolumab was not associated with a significant improvement in progression-free survival (PFS) or OS compared with chemotherapy among patients with previously untreated stage IV or recurrent PD-L1-positive NSCLC [25]. However, in patients with a high TMB, response rate was higher with nivolumab and median PFS was prolonged (9.7 vs. 5.8 months; hazard ratio [HR] for disease progression or death, 0.62; 95% CI: 0.38–1.00). There was no significant association between TMB and PD-L1 tumour expression level but the highest response rate was seen in patients with both high TMB and PD-L1 expression ≥50%.

Blood-based TMB can also be used as a biomarker to predict clinical efficacy of anti-PD-L1, atezolizumab. Data from the OAK and POPLAR trials of atezolizumab as second-line treatment of NSCLC reported improved PFS and OS versus docetaxel observed at a range of blood TMB cutpoints [26].

Whole exome DNA sequencing and copy number array data showed a landscape of significant alterations to genes and pathways responsible for maintaining DNA integrity in NSCLC. Tumours with nonsense mutations, indels, or homozygous deletions in the FANCE or MLH1 genes have significantly higher TMB [27]. Smoking was not a sufficient substitute biomarker for TMB in NSCLC, although DNA polymerase and mismatch excision repair pathway inactivation is enriched in smoking-sensitive NSCLC.

Comprehensive genomic analysis of tumours and hosts should lead to new insights into the host-tumour interaction, new biomarkers for selection of patients, and potentially new therapeutic approaches. This will require the collection of biospecimens and intensive, rational, scientific analysis of specimens before and during treatment.

## Immunotherapy for advanced NSCLC: state of the art

Several PD-1/PD-L1 inhibitors are approved or under development for the treatment of NSCLC. In the phase II ATLANTIC study in heavily pre-treated patients with locally advanced or metastatic NSCLC, overall response rate (ORR) with anti-PD-L1 durvalumab was 7.5% (95% CI: 3.1–14.5) in patients with PD-L1 expression <25% and 16.4% (95% CI:10.8–23.5) in patients with PD-L1 expression ≥25% [28]. An ORR of 30.9% (95% CI: 20.2–43.3) was observed in patients with PD-L1 expression ≥90%. Durvalumab showed a manageable safety and tolerability profile, with most adverse events low-grade and resolved with treatment delay and/or immunosuppressive interventions. However, in the phase III MYSTIC trial (NCT02453282), the combination of first-line treatment with durvalumab and tremelimumab in previously untreated NSCLC patients did not meet the primary endpoint of improved PFS compared to standard of care in patients with ≥25% PD-L1 tumour expression (www.ascopost.com/News/57874). Moreover, durvalumab monotherapy did not show a PFS benefit over standard of care. Impressive results have been obtained with durvalumab in locally advanced disease in the PACIFIC trial (NCT02125461) regardless of PD-1 expression. After induction chemotherapy, patients receiving concurrent chemo-radiotherapy were randomized to durvalumab maintenance (n=476) or placebo (n=237) for up to 12 months. The primary study endpoint was reached with a median PFS of 16.8 months for durvalumab and 5.6 months for placebo arms (HR 0.52) [29].

In the OAK study, 1225 patients with previously treated NSCLC were stratified according to PD-L1 status, number of prior chemotherapy regimens and histology before being randomised to anti-PD-L1 atezolizumab 1200 mg or docetaxel 75 mg/m2 every 3 weeks [30]. In a preliminary analysis of data from 850 patients, there was a 27% improvement in OS in the atezolizumab group compared with docetaxel (p=0.0003), regardless of PD-L1 expression levels and including patients with PD-L1 expression <1%. When patients were stratified according to their PD-L1 expression level, OS was 59% greater among patients in the highest tertile of PD-L1 expression with atezolizumab compared with docetaxel (p<0.0001). However, even in patients with no PD-L1 expression, there was a significant 25% improvement in OS with atezolizumab compared to docetaxel. Improvements in OS were similar in patients with squamous and non-squamous histology. Atezolizumab was well tolerated with a favourable safety profile. Atezolizumab is currently being assessed as first-line monotherapy or combined with chemotherapy in several trials in patients with squamous or non-squamous NSCLC. It has been reported that a phase III trial atezolizumab combined with bevacizumab plus chemotherapy met its primary endpoint of PFS versus bevacizumab plus chemotherapy as first-line therapy of patients with non-squamous NSCLC (https://www.roche.com/media/store/releases/med-cor-2017-12-07.htm).

The PD-1 inhibitors pembrolizumab and nivolumab have both shown efficacy in NSCLC. In the KEYNOTE-010 trial, pembrolizumab prolonged OS and had a favourable benefit-to-risk profile in patients with previously treated PD-L1-positive advanced NSCLC [31], while in KEYNOTE-024, pembrolizumab was superior to chemotherapy as first-line therapy for advanced NSCLC with PD-L1 tumour expression ≥50% [32]. The combination of pembrolizumab with carboplatin and pemetrexed has also been shown to be an effective and tolerable first-line treatment option for patients with advanced non-squamous NSCLC, with longer PFS versus carboplatin and pemetrexed in a phase II randomized trial (HR 0.49, 95% CI: 0.29–0.83, p=0.0035) [33]. First-line pembrolizumab in combination with pemetrexed and either cisplatin or carboplatin is also being assessed in non-squamous NSCLC in the phase III KEYNOTE-189 trial (NCT02578680). Recently the manufacturer reported that the study met its primary endpoints with pembrolizumab combined with chemotherapy improving PFS and OS compared to chemotherapy alone (http://investors.merck.com/news/press-release-details/2018/Mercks-KEYTRUDAR-pembrolizumab). Clinical trials have also suggested that nivolumab provides long-term clinical benefit and a favourable tolerability profile compared with docetaxel in previously-treated patients with advanced NSCLC [34]. In the CheckMate-026 trial, nivolumab was not associated with significantly improved PFS or OS when compared with platinum-based chemotherapy in patients with previously untreated NSCLC with a PD-L1 expression level ≥5% [25]. However, nivolumab improved ORR and PFS compared with platinum doublet chemotherapy in patients with high TMB. The results of a large phase III randomized trial (CheckMate 227; NCT02477826) assessing the role of nivolumab as a single agent, combined with chemotherapy or with ipilimumab in a first-line setting are pending.

## Immunotherapy for head and neck cancer

Immune checkpoint therapy, specifically PD-1 pathway blockade, improves survival in patients with metastatic squamous cell carcinoma of the head and neck (SCCHN) [35]. However, PD-1 monotherapy in SCCHN seems associated with relatively lower ORRs compared with other indications, such as NSCLC or melanoma, and many patients fail to respond. Cetuximab, an IgG1 isotype, is a standard of care treatment for locally advanced and recurrent and/or metastatic SCCHN. In addition to EGFR inhibition, cetuximab mediates clinically relevant antibody-dependent cell-mediated cytotoxicity (ADCC) and other immune activity in the intratumoural space [36]. Cetuximab can prime the immune system for checkpoint inhibitor therapy by recruiting cytotoxic cell effectors of both the innate and adaptive immune systems to the tumour [36]. However, associated negative feedback loops lead to immune checkpoint-mediated immunosuppression. Therefore, co-targeting of these immunosuppressive processes has the potential to improve patient outcomes, given the potential synergy between the different mechanisms of action of cetuximab and immunotherapy.

In the CheckMate-141 study, nivolumab resulted in significantly prolonged OS versus investigators’ choice of therapy (methotrexate, docetaxel, or cetuximab) in patients with platinum-refractory SCCHN. Nivolumab improved OS versus investigators’ choice regardless of prior cetuximab, although improvement was greater in patients without previous cetuximab treatment [37]. This may relate to cross-presentation of tumour antigens by DCs to T cells.

Several other trials of cetuximab and immune checkpoint inhibition combination therapy in SCCHN are ongoing. Cetuximab-mediated immune action drives crosstalk with a variety of immune cell types and processes, and therefore it holds the potential for combination with other immunotherapy agents, including motolimod, a TLR-8 agonist.

## Contrasts between immunotherapy for renal carcinoma and melanoma

Renal cell carcinoma (RCC) and melanoma have long been recognised as immune-responsive, with spontaneous remissions sometimes observed in both diseases. Cytokine-based treatment (*e.g.*, interferon [IFN], IL-2) only induced durable responses in a small fraction of both metastatic RCC and melanoma patients ([38, 39] However, checkpoint inhibitors targeting CTLA-4 and PD-1 have achieved durable responses in patients who were refractory to other therapies. In the CheckMate-025 trial that compared nivolumab with everolimus, the primary endpoint was met early on, with the nivolumab group achieving median OS of 25 months (95% CI: 21.8-not estimable) compared with 19.6 months (95% CI: 17.6–23.1 months) with everolimus [40]. Response rates in melanoma with single agent anti-PD1 inhibitors are higher than those seen in RCC.

Combination immunotherapy will become a new standard of care in RCC as it has in melanoma. In the CheckMate-214 trial, combined nivolumab plus ipilimumab followed by nivolumab monotherapy is being compared with sunitinib, a multi-targeted receptor tyrosine kinase inhibitor. At a minimum 17.5 months follow-up, confirmed ORR in intermediate/poor risk patients was 42% (9.4% CR) with nivolumab plus ipilimumab compared with 27% (1.2% complete responses [CR]) with sunitinib (p<0.0001) and median PFS was 11.6 versus 8.4 months (HR 0.82, p=0.0331) [41]. Interestingly no improvements have so far been seen in ORR or PFS in patients with a favourable risk. Low-risk, good prognosis metastatic RCC therefore appears to derive less benefit from combination immunotherapy than the equivalent group of melanoma patients. Further follow up is required to establish whether this is a real observation or whether a durable advantage with immunotherapy in this group emerges. A lower dose of ipilimumab (1 mg/kg) was administered in the RCC combination which is likely to be the reason for the good tolerability profile in RCC. Despite the lower ipilimumab dose, the activity of combination therapy in RCC is significantly greater than single agent PD1 blockade (cross-study comparison) suggesting a synergistic rather than additive effect, probably contributing to the contrasts between immunotherapy for RCC and melanoma.

## Dissecting the tumour microenvironment in renal cancer

RCC of clear-cell type (ccRCC), the most common type, has traditionally been considered an immune responsive tumour along with melanoma. Unlike melanoma, however, the mutation burden of ccRCC is modest (1–2 mutations per Mb). Immune responsiveness has been attributed to more antigenic mutations, reactivation of endogenous retroviruses, and a high level of inflammation. In regards inflammation, gene expression analyses have shown that ccRCC is a particularly inflamed tumour compared to other tumour types. Notably, several indicators of inflammation such as thrombocytosis, neutrophilia and anaemia are established prognostic factors in metastatic ccRCC. Furthermore, there appears to be a relationship between these prognostic variables and response to immunotherapy. Specifically, it has been shown in a phase 3 trial (Checkmate-214) that ipilimumab/nivolumab is superior to sunitinib in patients with intermediate and poor risk disease (with at least one risk factor including the aforementioned plus hypercalcemia, poor performance status and time to systemic therapy of less than one year), but not in those in a good risk group [41]. Thus, understanding how ccRCC induces inflammation may help identify determinants of immune responsiveness.

To understand how ccRCC induces inflammation, we sought to probe the relationship between the tumour and the tumour microenvironment (TME). Different approaches have been previously explored including both experimental and *in silico* approaches to evaluate the TME. Experimental approaches have focused on single cell analyses using mass cytometry or single cell RNA sequencing [42, 43]. *In silico* approaches have attempted to deconvolute the bulk tumour gene expression signature to distinguish contributions from the tumour cells versus its microenvironment [44]. By using previously characterized cell type-specific gene expression signatures, their presence in the TME can be ascertained.

To explore the ccRCC TME, we have taken a novel approach. To separate from bulk tumour gene expression the tumour and TME components, we leveraged tumorgrafts (TGs or PDX models). Over the years, we have implanted kidney tumour samples from over 1000 patients orthotopically in mice. The most aggressive of these samples will grow to form large tumours within a few weeks. These tumours are made up of human tumour cells, but the stroma is from the mouse host [45–47]. We reasoned that by subtracting the human tumorgraft signature from the corresponding original patient’s tumour, we would be left with a gene expression signature corresponding to the TME. This signature we refer to as the empiric tumour microenvironment signature or eTME. We applied this approach to 35 RCCs, including 29 ccRCCs, for which we performed RNAseq. We subtracted from the patient bulk tumour RNAseq signature, the corresponding TG signature (human genes only) and the signature from the particular patient normal kidney. To accomplish this, we developed an algorithm, dissecting heterogeneous tumours (DisHet), which is based on a Bayesian approach. DisHet identified over 2000 genes expressed at ≥3-fold higher levels in the TME than in RCC, including >900 genes expressed at >20 fold higher levels [unpublished data]. The majority of these genes have not been previously associated with the RCC TME. Interestingly, the eTME signature was able to resolve different RCC histologies to an even greater extent than traditional bulk gene expression signatures. Furthermore, we identified an inflamed gene expression signature that was associated with anaemia, thrombocytosis and poor patient survival.

## What role for immunotherapy in the treatment of hepatocellular carcinoma?

The advent of sorafenib improved survival outcomes among patients with HCC. Recent studies have demonstrated the efficacy of new targeted agents; first-line lenvatinib (a tyrosine kinase inhibitor) has been shown to be non-inferior to sorafenib [48], and regorafenib (another tyrosine kinase inhibitor) is used after sorafenib failure [49]. The potential role of immune checkpoint inhibitors is also a focus of attention. HCC is typically an inflammation-associated cancer and can be immunogenic. Hepatitis C (HCV) and hepatitis B (HBV) infection have been associated with upregulation of PD-1, and upregulation of PD-1 and PD-L1 in HCC is associated with poor outcomes. PD-1 blockade with nivolumab may boost host immunity against HCC and improve clinical outcomes.

In the phase I/II CheckMate-040 trial in patients with advanced HCC with or without HCV or HBV, nivolumab had a manageable safety profile with no maximum tolerated dose reached in a dose escalation phase [50]. In the dose expansion phase, nivolumab 3 mg/kg resulted in an ORR of 20% (95% CI: 15–26), with 18% partial responses (PR) and 1% CR. Disease control rate was 64% (95% CI: 58–71) and OS at 9 months was 74% (95% CI: 67–79). Response was not correlated with tumour PD-L1 expression. Further trials of anti-PD agents in HCC are ongoing. These include nivolumab versus sorafenib as first-line treatment in patients (NCT02576509), and second-line pembrolizumab compared with best supportive care (NCT02702401).

## Dendritic cell vaccine combinations for melanoma

DCs play a critical role in promoting an immune response against antigens, including TAAs. DC are capable of boosting a memory T cell response and are effective initiators of naïve T cell responses. DC vaccines were originally considered a stand-alone therapeutic approach to promote regression of tumours. However, with the advent of immune checkpoint inhibitors and data supporting the need for a pre-existing immune response in the tumour for successful checkpoint blockade response, vaccines may have a role in promoting anti-tumour immune responses in patients who lack spontaneous immunity.

Although the identification of an optimal antigen is important for directing anti-tumour immunity to the tumour, the spread of the immune response from one antigen to another antigen expressed in the same tissue (‘determinant’ [or epitope or antigen] spreading) has also been associated with superior clinical outcome [51]. AdVTMM2, an E1/E3-deleted adenovirus encoding three full length melanoma antigens (tyrosinase, MART-1 and MAGE-A6), expresses mRNA and protein for all antigens, and AdVTMM-transduced DCs activate both CD8^+^ and CD4^+^ T cells that recognise melanoma tumour cells more efficiently than single antigen adenovirus-DC vaccines [51]. Addition of physiological levels of IFN-α can further amplifies melanoma antigen-specific T cell activation *in vitro*. NK cells can also be recruited by AdV-transduced DC via IL-8 and IFN-γ-inducible protein-10 (IP-10), and show cytotoxic activity. These data formed the hypotheses tested in a recent clinical trial.

Melanoma patients were found to have decreased expression of co-stimulatory molecules (e.g. ICOSL, OX40L) with AdVTMM2 DC compared to healthy donors, while both melanoma patients and healthy donors had increased expression of co-inhibitory molecules (TIM3L, PD-L1, PD-L2, CTLA-4) in matured compared with immature DC [unpublished data]. DC vaccination successfully increased the frequency of functional melanoma-specific CD8 and CD4 T cells, and approximately 7–10% of tumour-specific CD8 cells were CTLA-4 positive while 20–28% were PD-1 positive. Melanoma patient-derived AdVTMM2 DC were also inferior at producing chemokines and produce more immunosuppressive cytokines compared to DC vaccines made from healthy donor cells. Addition of one month of high dose systemic IFN-α did not improve T cell responses or clinical responses. IFN-α therapy did increase expression of chemokine receptors on the three NK cell subsets and increases circulating NK cell lytic ability. These data support clinical testing of new rational combinations with antigen loaded DC, such as with checkpoint blockade.

## New developments in oncolytic virus immunotherapy

Oncolytic viruses mediate anti-tumour activity via multiple mechanisms of action and are uniquely positioned to serve as the foundation for combination immunotherapy regimens. Herpes simplex virus type-1 (HSV-1) mediates immunogenic oncolysis, has a broad host cell range, induces innate and adaptive immune responses that can encode eukaryotic transgenes, is genetically stable and replication competent, and is easy to attenuate [52]. Talimogene laherparepvec (T-VEC) is an intralesional oncolytic virus therapy based on a modified HSV-1. T-VEC selectively targets tumour cells, causing regression in injected lesions and inducing immunologic responses that mediate regression at uninjected/distant sites. T-VEC has shown synergy with ipilimumab and pembrolizumab in melanoma, without additional toxicity [53, 54].

The next generation of HSV-1 oncolytic viruses are now being developed. These are based on a very potent underlying HSV strain with an additional increase in direct tumour cell killing and viral spread through expression of a fusogenic protein that provides a 10–100-fold improvement in lytic activity *in vitro* and highly immunogenic cell death and release of tumour antigen-containing exosomes. Expression of Gibbon Ape Leukaemia virus (GALV) provides enhanced potency in human tumour cell lines, promoting virus distribution within the tumour microenvironment while GM-CSF expression stimulates DC activity in the tumour [unpublished data]. This platform (RP1) can then be used to deliver additional potent immune stimulatory proteins directly to the tumour, focusing on pathways where systemic engagement is sub-optimal. RP1 has been shown to reduce large injected and uninjected rat 9L glioma tumours in immune-competent rats and a phase I/II clinical trial of RP1 alone and in combination with checkpoint blockade across several tumour types has been initiated. RP2 and RP3 are derivatives of RP1 that express additional proteins. RP2 expresses an anti-CTLA-4 antibody-like molecule and RP3 additionally expresses optimised immune co-stimulatory pathway ligands. These therapeutics provide targeted delivery to the sites of immune response initiation in the tumour and draining lymph nodes with the goal of focusing systemic immune-based efficacy on tumours and limiting off-target toxicity.

An important consideration is the need for biomarkers for oncolytic virus immunotherapy. Oncolytic viruses are able to induce T cell recruitment and activation and increase PD-L1 expression within the tumour microenvironment. For example, T-VEC and pembrolizumab increased CD8^+^ T cell density and PD-L1 in responding lesions [54]. T-VEC and pembrolizumab also induced type 1 IFN and PD-L1 expression [54]. Oncolytic viruses can also result in expanded neoantigen-specific CD8^+^ T cells in PD-1 refractory tumour cells. Stimulator of IFN genes (STING) is an essential molecule that controls the production of host defence proteins, including type I IFNs and proinflammatory cytokines [55]. STING expression correlates with tumour cell resistance to T-VEC infection [56]. STING transduction in STING-lo SK-Mel 2 cell lines inhibits lytic activity *in vitro* and STING knockout via CRSP/Cas9 restores lytic activity in resistant LOXIMVI melanoma cell lines. Anti-viral machinery status may thus be a potential biomarker for response.

## Building the next generation of highly potent adoptive cancer immunotherapies

Progress in gene engineering has resulted in an exponential growth in the number of adoptive cell transfer (ACT) trials [57]. Adoptive transfer of receptor-engineered T cells has produced impressive results in treating patients with B cell leukaemia and lymphoma. However, there is a need to enhance the efficacy of adoptive immunotherapies for the treatment of advanced solid cancers. Across clinical trials, there has been an association between T cell expansion and persistence and tumour regression in patients with hematologic and solid cancers receiving ACT. Thus, T cell persistence has been one of the few consistent biomarkers of response across the majority of ACT clinical trials [58, 59]. Thus, in theory, disruption of pathways that impair T cell persistence could result in enhanced anti-tumour efficacy following ACT. Identifying potentially actionable factors that limit the intrinsic capacity of T cells to expand and persist could improve outcomes and is not an approach that is addressed with current therapeutic options.

The majority of human cancers were found to overexpress the gene encoding Fas-ligand (FasL) relative to normal tissues. T cells used for clinical ACT are skewed towards antigen experienced subsets, all of which constitutively co-express Fas/CD95 [60]. Consequently, adoptively transferred T cells are poised to undergo apoptosis upon entering the tumour microenvironment. FasL-induced apoptosis requires both Fas oligomerisation and FADD recruitment. Overexpression of a Fas death domain variant prevents Fas crosslinking. T cells engineered with Fas mutants are expressed 5–10-fold higher compared with wild-type Fas, preventing FasL-induced apoptosis in a dominant negative fashion. T cells engineered with a Fas dominant negative receptor (DNR) exhibited superior *in vivo* persistence and show superior anti-tumour efficacy in animal models. Fas DNRs block Fas-induced AKT pathway activation and limit AKT-induced T cell differentiation [61]. CD8+ T cell differentiation status is highly correlated with anti-tumour efficacy in mice. Normalising for T cell differentiation does not compromise the *in vivo* anti-tumour efficacy of Fas DNR modified T cells. Germline loss of Fas function can result in an autoimmune lymphoproliferative syndrome (ALPS); however, mice receiving ACT of Fas modified T cells do not develop an acquired ALPS syndrome and human T cells modified with Fas DNR are protected from FasL induced T cell death. In summary, T cells engineered to intrinsically resist FasL-induced cell death have superior anti-tumour efficacy and represent a potentially universal strategy to enhance adoptive immunotherapy for advanced solid cancers.

## A novel human memory CD4 T cell subset with durable anti-tumour properties

Although CD8+ T cells have shown clinical promise, human CD4+ T cell subsets that exhibit properties of stemness and natural migration to the tumour have yet to be identified. Previous studies on CD4+ T cells has shown that they polarize to a type 17 phenotype (Th17) that exhibits stem cell-like memory qualities and yield greater tumour regression and persistence *in vivo* than other T helper subsets. The hypothesis that human Th17 cells are most effective has prompted investigations to determine if genetically redirected human IL-17+ T cells with antigen receptors can mediate superior regression of human tumours than unpolarised redirected cells.

Co-stimulation has also been reported to impact the antitumor fate of Th17 cells. ICOS is a CD28 family costimulatory molecule that is structurally and functionally related to CD28 and CTLA-4. CD28 but not ICOS induces IL-2 and combining CD28 and ICOS augments the effector function of murine T cells. ICOS has been demonstrated to promote robust Th17-mediated immunity in melanoma and in mesothelioma [62, 63]. Interestingly, more recent investigations reveal that ICOS can promote T cells that have high expression of CD26 on their cell surface (CD26_high_) and which produce abundant IL-17 [unpublished data].

**Fig. 4:**
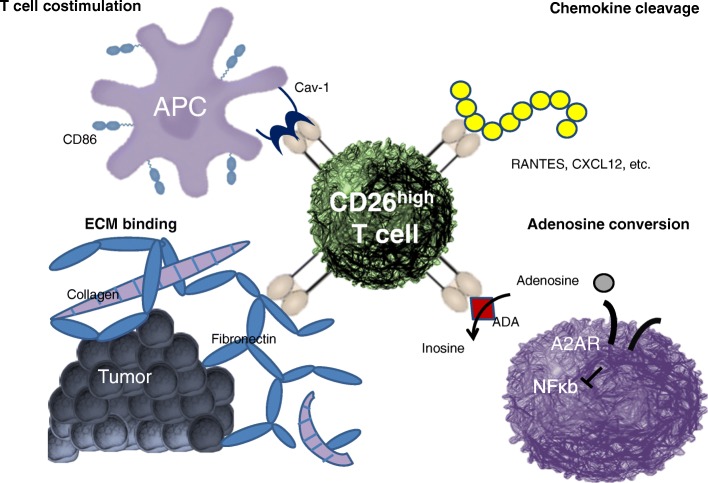
CD26 is an enzymatic, multifunctional protein

CD26 is an enzymatic, multifunctional protein shown to have a role in T cell co-stimulation as well as the binding of extracellular matrix proteins/adenosine deaminase (Fig. 4). CD26 expression correlates with specific CD4+ T cell subsets with distinct immunological properties. CD26_neg_ T cells possess a regulatory phenotype, but CD26_int_ T cells are mainly naïve and CD26_high_ T cells appear terminally differentiated and exhausted. Despite this, CD26_high_ T cells persist in and regress multiple solid tumours following ACT. Further analysis revealed that CD26_high_ cells have a rich chemokine receptor profile, profound cytotoxicity, resistance to apoptosis, and enhanced stemness [64].

In conclusion, CD26_high_ T cells have a mixed Th1/Th17/Th22 phenotype, yet are unique, are highly multifunctional and exert greater cytotoxicity and control tumour growth both *in vitro* and *in vivo*. These characteristics mean CD26_high_ T cells have a natural capacity to traffic to, regress and survive in solid tumours, properties that may help to improve cancer immunotherapy. This new finding is exciting because it means that CD26 could be targeted to augment adoptive T cell transfer therapy as well as other forms of cancer immunotherapy, including checkpoint inhibitors and cancer vaccines.

## Chimeric-antigen receptor T cells for haematological malignancies: building upon CAR-T19

Anti-CD19 chimeric-antigen receptor T cells (CART19) have shown high response rates and durable remissions in relapsed/refractory(r/r) B-cell acute lymphoblastic leukaemia (B-ALL) and non-Hodgkin lymphoma (NHL) [65]. Thanks to this impressive clinical activity two CART19 products, CTL019 (Kymriah®, Novartis) and KTE-19 (Yescarta®, Kite Pharma/Gilead) were recently approved by the US Food and Drug Administration for the treatment of refractory or relapsed paediatric and young-adult B-ALL or refractory or relapsed adult diffuse large-B cell lymphoma, respectively. However, despite the high response rates after CART19 immunotherapy, a subset of patients still relapses and, in particular in B-ALL, the majority of the relapses are caused by the loss of CD19 on leukemic cells [66]. Several mechanisms of CD19-targeted therapy resistance have been described, including convergence of acquired mutations of the CD19 gene, alternative CD19 splicing [67] and others [68, 69]. However, these known mechanisms do not explain all cases of CD19-negative B-ALL relapses. We recently reported [70] the case of a paediatric B-ALL patient who relapsed 9 months after CTL019 with a CD19-negative leukaemia. Unexpectedly, 100% of leukemic blasts were found to aberrantly express the CAR19 protein on the surface. Our data show that a single leukemic cell was accidentally transduced with CAR19, survived the 10-day manufacturing process and, upon reinfusion into the patient, was solely responsible for the relapse 9 months later. The relapsed clone was resistant to killing by CART19 cells in a xenograft model yet retained sensitivity to anti-CD22 CAR-T cells. Moreover, as the high expression of the CAR19 in leukemic cells and its absence from normal tissues make it an ideal engineered tumour target, we developed anti-CAR19 CAR T cells with the goal of specifically targeting CAR19+ B-ALL [71]. We proved that anti-CAR19 CART can efficiently target CAR19+ leukaemia in xenograft models, thereby presenting an opportunity for specific targeting without off-target toxicity. In conclusion, highly targeted and potent immunotherapies can lead to novel specific relapse mechanisms and only a deep understanding of the pathogenesis of these relapses can drive the generation of new tailored treatments.

## System immunology to decipher the tumour microenvironment

Systems biology approaches have facilitated analysis of the complex interaction between tumours and the host-immune response and have allowed the definition of the immune contexture (*i.e.* the type, density and location of cytotoxic and memory T cells within the tumour), which can be a strong prognostic marker when quantified by the Immunoscore [72, 73]. Tumour progression, invasion and recurrence are dependent on the immune contexture and Immunoscore and survival is strongly influenced by pre-existing immunity. The tumour microenvironment evolves with tumour progression, with the immune infiltrate composition changing at each tumour stage and particular cells having a major impact on survival. In an analysis of the spatiotemporal dynamics of 28 different immune cell types infiltrating tumours, T follicular helper and innate cells increased, whereas most T cell densities decreased with tumour progression [74]. The number of B cells increased at a late stage and showed a dual effect on recurrence and progression. The genetic features of the tumour contribute to shaping the tumour escape mechanisms. In hypermutated tumours, immune-inhibitors are upregulated and immunosuppressive cells are depleted, while in non-hypermutated tumours, immune-inhibitors are downregulated but immunosuppressive cells are enriched [75].

The tumour microenvironment and immune contexture are critical determinants of dissemination to distant metastases [76]. However, a key question is whether an immune escape occurs at the metastatic stage. In a study to assess how the metastatic immune landscape impacts response to treatment and patients’ outcome, Immunoscore was analysed after complete curative resection of multiple metastases at different sites (n = 441) in patients with colorectal cancer [77]. Response to treatment and prolonged survival were significantly associated with high-immune densities quantified into the least immune-infiltrated metastasis. Tumours with a pathological response had higher densities of immune cells (CD3, CD8, CD20) and patients who responded to neoadjuvant treatment had higher densities of immune cells (CD3, CD8, CD20, CD45RO, FOXP3). Immunoscore more than tumour regression predicted PFS and OS and surpassed all known variables associated with outcome in stage IV disease. High Immunoscore within metastases predicted prolonged survival and the least regressing metastasis had the lowest Immunoscore. Immunoscore also predicted OS and long-term survival in patients with brain metastases and was independent from established prognostic parameters [78].

## Conclusions

The immunotherapy of cancer has made rapid and major advances in recent years and is now recognised as a critical element in the treatment of many cancer types. Increased understanding of the complex interactions between tumours and the host immune response is leading to the development of novel therapeutic strategies across different cancers. In particular, research into a wide range of different and potentially synergistic immunotherapy combinations is ongoing and may hopefully lead to more durable responses for higher numbers of patients. The development and utilisation of effective biomarkers to guide the use of immunotherapies is another critical area for research and should help ensure that patients are treated with the most appropriate option. Novel immuno-based therapeutic approaches have already revolutionised cancer treatment and improved long-term outcomes for many patients and insights from ongoing and planned research should help to continue this progress.

## References

[1] Feng Z, Bethmann D, Kappler M, Ballesteros-Merino C, Eckert A, Bell RB, et al. Multiparametric immune profiling in HPV- oral squamous cell cancer. JCI Insight. 2017;2. pii: 93652. 10.1172/jci.insight.93652 PMC5518563 28724788

[2] Twitty CG, Jensen SM, Hu HM, Fox BA (2011). Tumor-derived autophagosome vaccine: induction of cross-protective immune responses against short-lived proteins through a p62-dependent mechanism. Clin Cancer Res..

[3] Sanborn RE. Randomized Ph II trial of allogeneic DPV-001 cancer vaccine alone or with adjuvant for curatively-treated stage III NSCLC (ID 4640). WCLC, 2016. P2.02-043.

[4] Fox BA, Boulmay BC, Li R, Happel KT, Paustian C, Moudgil TL. T cell population expansion in response to allogeneic cancer vaccine alone (DPV-001) or with granulocyte-macrophage colony-stimulating factor (GM-CSF) or imiquimod (I) for definitively-treated stage III NSCLC patients (pts). J Clin Oncol. 2017;35(15_suppl) 10.1200/JCO.2017.35.15_suppl.e14639.

[5] Jensen SM, Maston LD, Gough MJ, Ruby CE, Redmond WL, Crittenden M (2010). Signaling through OX40 enhances anti-tumor immunity. Semin Oncol..

[6] Butterfield LH, Ribas A, Meng WS, Dissette VB, Amarnani S, Vu HT (2003). T-cell responses to HLA-A*0201 immunodominant peptides derived from alpha-fetoprotein in patients with hepatocellular cancer. Clin Cancer Res..

[7] Butterfield LH, Ribas A, Dissette VB, Lee Y, Yang JQ, De la Rocha P (2006). A phase I/II trial testing immunization of hepatocellular carcinoma patients with dendritic cells pulsed with four alpha-fetoprotein peptides. Clin Cancer Res..

[8] Bray SM, Vujanovic L, Butterfield LH (2011). Dendritic cell-based vaccines positively impact natural killer and regulatory T cells in hepatocellular carcinoma patients. Clin Dev Immunol..

[9] Lee WC, Wang HC, Hung CF, Huang PF, Lia CR, Chen MF (2005). Vaccination of advanced hepatocellular carcinoma patients with tumor lysate-pulsed dendritic cells: a clinical trial. J Immunother..

[10] Palmer D, Midgley RS, Mirza N, Torr EE, Ahmed F, Steele J (2009). A phase II study of adoptive immunotherapy using dendritic cells pulsed with tumor lysate in patients with hepatocellular carcinoma. Hepatology..

[11] El Ansary M, Mogawer S, Elhamid SA, Alwakil S, Aboelkasem F, Sabaawy HE (2013). Immunotherapy by autologous dendritic cell vaccine in patients with advanced HCC. J Cancer Res Clin Oncol..

[12] Greten TF, Forner A, Korangy F, N’Kontchou G, Barget N, Ayuso C (2010). A phase II open label trial evaluating safety and efficacy of a telomerase peptide vaccination in patients with advanced hepatocellular carcinoma. BMC Cancer..

[13] Mizukoshi E, Nakagawa H, Kitahara M, Yamashita T, Arai K, Sunagozaka H (2015). Phase I trial of multidrug resistance-associated protein 3-derived peptide in patients with hepatocellular carcinoma. Cancer Lett..

[14] Sawada Y, Yoshikawa T, Ofuji K, Yoshimura M, Tsuchiya N, Takahashi M (2016). Phase II study of the GPC3-derived peptide vaccine as an adjuvant therapy for hepatocellular carcinoma patients. Oncoimmunology..

[15] Buonaguro L, HEPAVAC Consortium (2017). New vaccination strategies in liver cancer. Cytokine Growth Factor Rev..

[16] Snyder A, Makarov V, Merghoub T, Yuan J, Zaretsky JM, Desrichard A (2014). Genetic basis for clinical response to CTLA-4 blockade in melanoma. N Engl J Med..

[17] Fourcade J, Sun Z, Benallaoua M, Guillaume P, Luescher IF, Sander C (2010). Upregulation of Tim-3 and PD-1 expression is associated with tumor antigen–specific CD8+ T cell dysfunction in melanoma patients. J Exp Med..

[18] Chauvin J-M, Pagliano O, Fourcade J, Sun Z, Wang H, Sander C (2015). TIGIT and PD-1 impair tumor antigen-specific CD8+ T cells in melanoma patients. J Clin Invest..

[19] Danilova L, Wang H, Sunshine J, Kaunitz GJ, Cottrell TR, Xu H (2016). Association of PD-1/PD-L axis expression with cytolytic activity, mutational load, and prognosis in melanoma and other solid tumors. Proc Natl Acad Sci U S A..

[20] Westphal M, Lamszus K (2015). Circulating biomarkers for gliomas. Nat Rev Neurol..

[21] Lee JH, Long GV, Boyd S, Lo S, Menzies AM, Tembe V (2017). Circulating tumour DNA predicts response to anti-PD1 antibodies in metastatic melanoma. Ann Oncol..

[22] Sade-Feldman M, Jiao YJ, Chen JH, Rooney MS, Barzily-Rokni M, Eliane JP (2017). Resistance to checkpoint blockade therapy through inactivation of antigen presentation. Nat Commun..

[23] György B, Szabó TG, Pásztói M, Pál Z, Misják P, Aradi B (2011). Membrane vesicles, current state-of-the-art: emerging role of extracellular vesicles. Cell Mol Life Sci..

[24] Rizvi NA, Hellmann MD, Snyder A, Kvistborg P, Makarov V, Havel JJ (2015). Cancer immunology. Mutational landscape determines sensitivity to PD-1 blockade in non-small cell lung cancer. Science..

[25] Carbone DP, Reck M, Paz-Ares L, Creelan B, Horn L, Steins M (2017). First-line nivolumab in stage IV or recurrent non-small-cell lung cancer. N Engl J Med..

[26] Gandara DR, Kowanetz M, Mok TYSK, Rittmeyer A, Fehrenbacher L, Fabrizio D (2017). Blood-based biomarkers for cancer immunotherapy: tumor mutational burden in blood (bTMB) is associated with improved atezolizumab (atezo) efficacy. Ann Oncol.

[27] Sharpnack M, Cho JH, Oezkan F, Koenig M, Kim I, Otterson G, et al. The landscape of alteration of DNA integrity-related genes and their association with tumor mutation burden in non-small cell lung cancer. WCLC 2017, OA 18.02 https://s3.amazonaws.com/iaslc/pdf/WCLC2017_Abstract_Book_Web.pdf

[28] Garassino MC, Vansteenkiste JF, Kim J. Durvalumab in ≥3rd-line locally advanced or metastatic, EGFR/ALK wild-type NSCLC: results from the phase 2 ATLANTIC study. J Thoracic Oncol 2017;12(1 Suppl):S10–S11 Abstract PL04a.03.

[29] Antonia SJ, Villegas A, Daniel D, Vicente D, Murakami S, Hui R (2017). Durvalumab after chemoradiotherapy in stage III non-small-cell lung cancer. N Engl J Med..

[30] Barlesi F, Park K, Ciardiello F, von Pawel J, Gadgeel S, Hida T, et al. Primary analysis from OAK, a randomized phase III study comparing atezolizumab with docetaxel in 2L/3L NSCLC. Ann Oncol 2016;27(suppl 6): LBA44 PR.

[31] Herbst RS, Baas P, Kim DW, Felip E, Pérez-Gracia JL, Han JY (2016). Pembrolizumab versus docetaxel for previously treated, PD-L1-positive, advanced non-small-cell lung cancer (KEYNOTE-010): a randomised controlled trial. Lancet..

[32] Reck M, Rodríguez-Abreu D, Robinson AG, Hui R, Csőszi T, Fülöp A (2016). Pembrolizumab versus chemotherapy for PD-L1-positive non-small-cell lung cancer. N Engl J Med..

[33] Papadimitrakopoulou V, Gadgeel SM, Borghaei H, Gandhi L, Patnaik A, Powell SF (2017). First-line carboplatin and pemetrexed (CP) with or without pembrolizumab (pembro) for advanced nonsquamous NSCLC: Updated results of KEYNOTE-021 cohort G. J Clin Oncol.

[34] Horn L, Spigel DR, Vokes EE, Holgado E, Ready N, Steins M (2017). Nivolumab versus docetaxel in previously treated patients with advanced non-small-cell lung cancer: two-year outcomes from two randomized, open-label, phase III trials (CheckMate 017 and CheckMate 057). J Clin Oncol..

[35] Ferris RL, Blumenschein G, Fayette J, Guigay J, Colevas AD, Licitra L (2016). Nivolumab for recurrent squamous-cell carcinoma of the head and neck. N Engl J Med..

[36] Ferris RL, Lenz HJ, Trotta AM, García-Foncillas J, Schulten J, Audhuy F (2018). Rationale for combination of therapeutic antibodies targeting tumor cells and immune checkpoint receptors: Harnessing innate and adaptive immunity through IgG1 isotype immune effector stimulation. Cancer Treat Rev..

[37] Ferris RL, Licitra L, Fayette J, Even C, Blumenschein GR, Harrington K (2017). Nivolumab (Nivo) vs investigator’s choice (IC) in patients with recurrent or metastatic (R/M) squamous cell carcinoma of the head and neck (SCCHN): Efficacy and safety in CheckMate 141 by prior cetuximab use. J Clin Oncol.

[38] Rosenblatt J, McDermott DF (2011). Immunotherapy for renal cell carcinoma. Hematol Oncol Clin North Am..

[39] Achkar T, Tarhini AA (2017). The use of immunotherapy in the treatment of melanoma. J Hematol Oncol..

[40] Motzer RJ, Escudier B, McDermott DF, George S, Hammers HJ, Srinivas S (2015). Nivolumab versus everolimus in advanced renal-cell carcinoma. N Engl J Med..

[41] Escudier B, Tannir NM, McDermott DF, Frontera OA, Melichar B, Plimack ER, et al. CheckMate 214: Efficacy and safety of nivolumab + ipilimumab (N+I) v sunitinib (S) for treatment-naïve advanced or metastatic renal cell carcinoma (mRCC), including IMDC risk and PD-L1 expression subgroups. Ann Oncol 2017;28 (suppl_5):mdx440.029.

[42] Kim KT, Lee HW, Lee HO, Song HJ, Jeong d E, Shin S (2016). Application of single-cell RNA sequencing in optimizing a combinatorial therapeutic strategy in metastatic renal cell carcinoma. Genome Biol..

[43] Chevrier S, Levine JH, Zanotelli VRT, Silina K, Schulz D, Bacac M, et al. An Immune Atlas of Clear Cell Renal Cell Carcinoma. Cell. 2017;169:736-749.e18. 10.1016/j.cell.2017.04.016 PMC5422211 28475899

[44] Şenbabaoğlu Y, Gejman RS, Winer AG, Liu M, Van Allen EM, de Velasco G (2016). Tumor immune microenvironment characterization in clear cell renal cell carcinoma identifies prognostic and immunotherapeutically relevant messenger RNA signatures. Genome Biol..

[45] Sivanand S, Peña-Llopis S, Zhao H, Kucejova B, Spence P, Pavia-Jimenez A, et al. A validated tumorgraft model reveals activity of dovitinib against renal cell carcinoma. Sci Transl Med. 2012;4:137ra75. 10.1126/scitranslmed.3003643 PMC3570965 22674553

[46] Peña-Llopis S, Vega-Rubín-de-Celis S, Liao A, Leng N, Pavía-Jiménez A, Wang S (2012). BAP1 loss defines a new class of renal cell carcinoma. Nat Genet..

[47] Pavía-Jiménez A, Tcheuyap VT, Brugarolas J (2014). Establishing a human renal cell carcinoma tumorgraft platform for preclinical drug testing. Nat Protoc..

[48] Kudo M, Finn RS, Qin S, Han KH, Ikeda K, Piscaglia F (2018). Lenvatinib versus sorafenib in first-line treatment of patients with unresectable hepatocellular carcinoma: a randomised phase 3 non-inferiority trial. Lancet..

[49] Bruix J, Qin S, Merle P, Granito A, Huang YH, Bodoky G (2017). Regorafenib for patients with hepatocellular carcinoma who progressed on sorafenib treatment (RESORCE): a randomised, double-blind, placebo-controlled, phase 3 trial. Lancet.

[50] El-Khoueiry AB, Sangro B, Yau T, Crocenzi TS, Kudo M, Hsu C (2017). Nivolumab in patients with advanced hepatocellular carcinoma (CheckMate 040): an open-label, non-comparative, phase 1/2 dose escalation and expansion trial. Lancet..

[51] Blalock LT, Landsberg J, Messmer M, Shi J, Pardee AD, Haskell R (2012). Human dendritic cells adenovirally-engineered to express three defined tumor antigens promote broad adaptive and innate immunity. Oncoimmunology..

[52] Harrington KJ, Puzanov I, Hecht JR, Hodi FS, Szabo Z, Murugappan S, Kaufman HL (2015). Clinical development of talimogene laherparepvec (T-VEC): a modified herpes simplex virus type-1-derived oncolytic immunotherapy. Expert Rev Anticancer Ther..

[53] Chesney JA, Puzanov I, Ross MI, Collichio FA, Milhem MM, Chen L (2017). Primary results from a randomized (1:1), open-label phase II study of talimogene laherparepvec (T) and ipilimumab (I) vs I alone in unresected stage IIIB- IV melanoma. J Clin Oncol.

[54] Ribas A, Dummer R, Puzanov I, VanderWalde A, Andtbacka RHI, Michielin O (2017). Oncolytic virotherapy promotes intratumoral T cell infiltration and improves anti-PD-1 immunotherapy. Cell..

[55] He L, Xiao X, Yang X, Zhang Z, Wu L, Liu Z (2017). STING signaling in tumorigenesis and cancer therapy: A friend or foe?. Cancer Lett..

[56] Xia T, Konno H, Barber GN (2016). Recurrent Loss of STING Signaling in Melanoma Correlates with Susceptibility to Viral Oncolysis. Cancer Res..

[57] Klebanoff CA, Rosenberg SA, Restifo NP (2016). Prospects for gene-engineered T cell immunotherapy for solid cancers. Nat Med..

[58] Maude S, Barrett DM (2016). Current status of chimeric antigen receptor therapy for haematological malignancies. Br J Haematol..

[59] Yu S, Li A, Liu Q, Li T, Yuan X, Han X, Wu K (2017). Chimeric antigen receptor T cells: a novel therapy for solid tumors. J Hematol Oncol..

[60] Klebanoff CA, Gattinoni L, Restifo NP (2012). Sorting through subsets: which T-cell populations mediate highly effective adoptive immunotherapy?. J Immunother..

[61] Klebanoff CA, Scott CD, Leonardi AJ, Yamamoto TN, Cruz AC, Ouyang C (2016). Memory T cell-driven differentiation of naive cells impairs adoptive immunotherapy. J Clin Invest..

[62] Paulos CM, Carpenito C, Plesa G, Suhoski MM, Varela-Rohena A, Golovina TN (2010). The inducible costimulator (ICOS) is critical for the development of human T(H)17 cells. Sci Transl Med..

[63] Majchrzak K, Nelson MH, Bowers JS, Bailey SR, Wyatt MM, Wrangle JM (2017). β-catenin and PI3Kδ inhibition expands precursor Th17 cells with heightened stemness and antitumor activity. JCI Insight..

[64] Bailey SR, Nelson MH, Majchrzak K, Bowers JS, Wyatt MM, Smith AS (2017). Human CD26^high^ T cells elicit tumor immunity against multiple malignancies via enhanced migration and persistence. Nat Commun..

[65] Rotolo A, Karadimitris A, Ruella M. Building upon the success of CART19: chimeric antigen receptor T cells for hematologic malignancies. Leuk Lymphoma. 2017:1–16. 10.1080/10428194.2017.1403024. 10.1080/10428194.2017.1403024 PMC6814196 29165008

[66] Ruella M, Maus MV (2016). Catch me if you can: Leukemia escape after CD19-directed T cell immunotherapies. Comput Struct Biotechnol J..

[67] Sotillo E, Barrett DM, Black KL, Bagashev A, Oldridge D, Wu G (2015). Convergence of acquired mutations and alternative splicing of CD19 enables resistance to CART-19 immunotherapy. Cancer Discov..

[68] Gardner R, Wu D, Cherian S, Fang M, Hanafi LA, Finney O (2016). Acquisition of a CD19 negative myeloid phenotype allows immune escape of MLL-rearranged B-ALL from CD19 CAR-T cell therapy. Blood..

[69] Jacoby E, Nguyen SM, Fountaine TJ, Welp K, Gryder B, Qin H (2016). CD19 CAR immune pressure induces B-precursor acute lymphoblastic leukaemia lineage switch exposing inherent leukaemic plasticity. Nat Commun..

[70] Lacey SF, Xu J, Ruella M, Barrett DM, Kulikovskaya I, Ambrose DE (2016). Cars in leukemia: relapse with antigen-negative leukemia originating from a single b cell expressing the leukemia-targeting CAR. Blood.

[71] Ruella M, Barrett DM, Shestova O, Perazzelli J, Posey AD, Kozlowski M (2017). A cellular antidote to specifically deplete Anti-CD19 chimeric antigen receptor (CAR19) positive cells. Blood..

[72] Galon J, Costes A, Sanchez-Cabo F, Kirilovsky A, Mlecnik B, Lagorce C (2006). Type, density, and location of immune cells within human colorectal tumors predict clinical outcome. Science.

[73] Galon J, Angell HK, Bedognetti D, Marincola F (2013). The continuum of cancer immunosurveillance: prognostic. predictive and mechanistic signatures. Immunity..

[74] Bindea G, Mlecnik B, Tosolini M, Kirilovsky A, Waldner M, Obenauf AC (2013). Spatiotemporal dynamics of intratumoral immune cells reveal the immune landscape in human cancer. Immunity..

[75] Angelova M, Charoentong P, Hackl H, Fischer ML, Snajder R, Krogsdam AM, Waldner MJ, Bindea G, Mlecnik B, Galon J, Trajanoski Z. Characterization of the immunophenotypes and antigenomes of colorectal cancers reveals distinct tumor escape mechanisms and novel targets for immunotherapy. Genome Biol. 2015;16(64) 10.1186/s13059-015-0620-6 PMC4377852 25853550

[76] Mlecnik B, Bindea G, Kirilovsky A, Angell HK, Obenauf AC, Tosolini M, et al. The tumor microenvironment and Immunoscore are critical determinants of dissemination to distant metastasis. Science Transl Med. 2016; 24;8(327):327ra26 10.1126/scitranslmed.aad6352 26912905

[77] Mlecnik B, Van den Eynde M, Bindea G, Church SE, Vasaturo A, Fredriksen T, et al. Comprehensive intrametastatic immune quantification and major impact of immunoscore on survival. J Natl Cancer Inst. 2018;110(1). 10.1093/jnci/djx123 28922789

[78] Berghoff AS, Fuchs E, Ricken G, Mlecnik B, Bindea G, Spanberger T, et al. Density of tumor-infiltrating lymphocytes correlates with extent of brain edema and overall survival time in patients with brain metastases. Oncoimmunology. 2016;5(1) 10.1080/2162402X.2015.1057388 PMC4760339 26942067

